# The use of liver biomechanics in forensic pathology

**DOI:** 10.1007/s00414-025-03581-4

**Published:** 2025-09-17

**Authors:** Johann Zwirner, Pavithran Devananthan, Natalia Kabaliuk, Paul D. Docherty, Benjamin Ondruschka

**Affiliations:** 1 Institute of Legal Medicine, University Medical Center Hamburg-Eppendorf, Hamburg, Germany; 2 Department of Oral Sciences, University of Otago, Dunedin, New Zealand; 3 Department of Mechanical Engineering, University of Canterbury, Christchurch, New Zealand; 4 Biomolecular Interaction Centre, University of Canterbury, Christchurch, New Zealand

**Keywords:** Forensic biomechanics, Human liver, Rheometry, Post-mortem interval, Time since death estimation

## Abstract

Stiffness and plasticity of human tissues are routinely assessed during forensic autopsy and have recently been identified as a promising metric for estimating time since death in animal models. In this study, the biomechanical state of the human liver is investigated concerning pathology, age at death, sex, liver weight, autolysis, and blood congestion. Additionally, its use for biomechanical time since death estimation is evaluated. The storage, loss, and complex shear moduli of 54 human liver parenchyma samples collected during routine forensic autopsies, were determined using a rheometer. All samples were microscopically analyzed for signs of pathology, autolysis, and blood congestion. High-grade fatty liver samples (*n* = 6) exhibited significantly higher storage moduli, and complex shear moduli compared to healthy (*n* = 27), low-grade fatty liver (*n* = 14), and cirrhotic (*n* = 7) samples (*p* ≤ 0.02). High-grade fatty liver samples also had significantly higher loss moduli compared to healthy and cirrhotic samples (*p* ≤ 0.04). The rheological properties of the human liver were unrelated to age at death (*p* ≥ 0.26), liver weight (*p* ≥ 0.13), and sex (*p* ≥ 0.32). Autolysis significantly increased the loss moduli of healthy liver samples (*p* = 0.01). Blood congestion significantly lowered the loss moduli of healthy (*p* = 0.03) and fatty (*p* < 0.01) samples, as well as storage moduli (*p* = 0.01), and complex shear moduli (*p* = 0.01) of fatty samples. A significant positive correlation between the post-mortem interval and the loss modulus was observed for healthy samples, if only samples without signs of blood congestion were included (*p* = 0.02; *n* = 9). When stored at 4 °C for an average of eight days post-mortem, liver biomechanics was significantly altered by fatty infiltration, autolysis, blood congestion, and the post-mortem interval, while liver weight, age at death, and sex had no relevant impact.

## Introduction

The subjective assessment of the stiffness and plasticity of internal organs is a routine part of every forensic and clinical autopsy. Some aspects of this assessment are rather straightforward, such as diagnosing a fatty liver if the tissue is firm and has a yellowish coloration. Other aspects that contribute to the tissue’s mechanical behaviour at autopsy are less obvious to diagnose. For example, blood congestion due to centralization of the blood prior to death can make internal organs feel stiffer, including the liver. It is challenging to detect the biomechanical effects of blood congestion in fatty livers, as two aspects that increase the stiffness of the tissue are present simultaneously. Additionally, the degradation rate of post mortem liver sample biomechanics remains undetermined. Furthermore, it is unclear how factors such as liver weight, sex, and age at death predispose the liver to a particular biomechanical state at autopsy, which could serve as indicators for forensic investigators for various diagnostic matters.

In particular, the change in rheological properties in relation to the post-mortem interval (PMI) is of forensic interest, as this information could be useful for estimating the time since death. Recently, the concept of biomechanical time since death estimation has been introduced, demonstrating the possibility of discriminating between different PMI intervals with excellent diagnostic ability in the animal model [1, 2]. Biomechanical time since death estimation aims to measure the post-mortem decay of tissues by defining cut-off values for rheological properties that indicate particular PMI intervals with optimal diagnostic ability. The advantage of this approach is that it can also be applied when only parts of the cadaver are available, such as in dismemberment cases, where some conventional PMI estimation techniques are impossible.

Liver tissue is promising for biomechanical time since death estimations, as it is relatively homogeneous, thereby facilitating the uniformity of samples taken from different individuals. From practical experience, liver tissue is fairly stable post-mortem, and biomechanical changes are likely to affect the tissue after conventional estimates for PMI are no longer beneficial, e.g., fixation of livor mortis and disappearance of rigor mortis.

The aims of this study are:

A) To objectify the subjective forensic assessment of the biomechanical state of the human liver at autopsy, including potential confounding factors such as pathological state, age at death, sex, liver weight, autolysis, and blood congestion.
B) To investigate the potential of human liver samples for biomechanical time since death estimation.

## Materials/methods

### Tissue collection and preparation

**Fig. 1:**
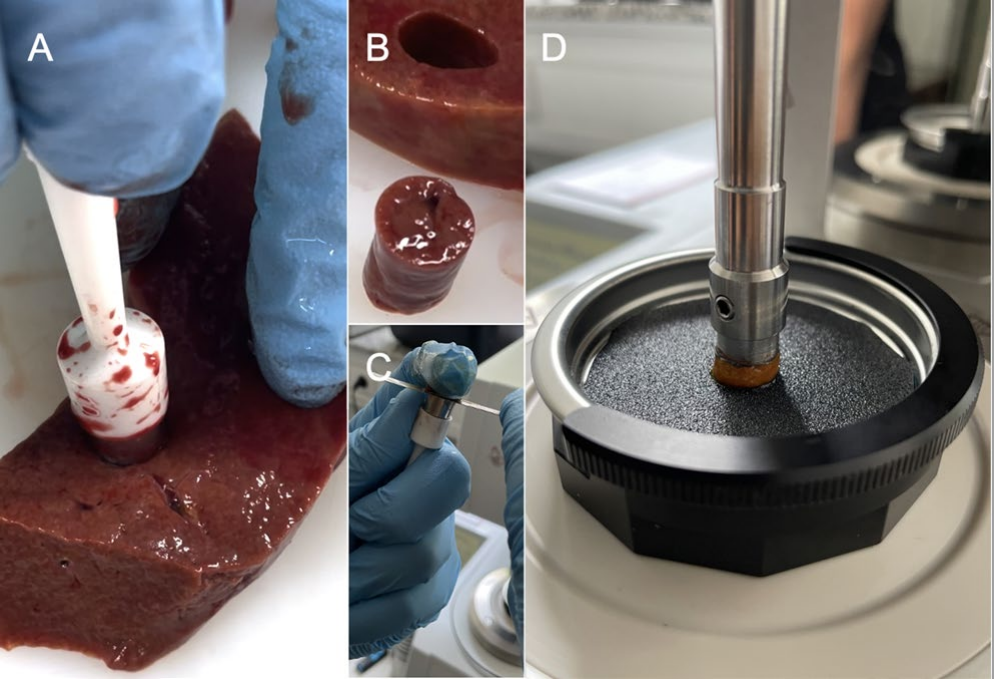
**(A)** The sample is punched from liver slices during autopsy. **(B)** Punching results in cylindric samples with a uniform diameter of 1 cm. **(C)** The sample height is uniformly trimmed to 5 mm using a customized punch and a metal blade. **(D)** The sample is shown loaded into the apparatus before saline solution is added

Human liver samples were taken from 54 different cadavers during routine forensic autopsies at the Institute of Legal Medicine of the University Medical Center Hamburg-Eppendorf. The study was approved by the Ethics Committee of the Hamburg Medical Association (reference number: 2024-101238-BO-ff). The samples were punched from 15 females and 39 males with an age at death of 60 ± 22 years (mean ± standard deviation) and an age range of 0 to 90 years (four children: 0, 2, 5, and 6 years). The PMI, defined as the time interval between death and time of autopsy for 50 of the cases, was 194 ± 89 h with a range of 42 to 341 h (4 had an unknown PMI). The cadavers were cooled to 4 °C immediately after arrival at the morgue and stored accordingly up until autopsy. Only cadavers with a rectal temperature of at least 30 °C upon arrival at the morgue were included in the PMI measurements to ensure a high level of comparability of the overall sample. Cadavers that were cooled before arrival at the morgue following death in a hospital with rapid transfer to a cooling unit were also included. The liver weight of the overall sample was 1528 ± 617 g with a range of 178 to 4125 g. Circular cylindrical parenchyma samples were punched from routinely prepared liver slices during autopsy using a biopsy punch with a diameter of 10 mm, excluding capsular components and large vessels, such as the main branches of the hepatic veins **(**Fig. 1A, B**)**. The sample height was trimmed to 5 mm using a customized biopsy punch with a depth of 5 mm and a sharp microtome blade to cut off the protruding tissue **(**Fig. 1C**)**. Following preparation, the samples were transferred to small lockable plastic containers, placed in a refrigerator at 4 °C, and tested within two hours after retrieval.

### Biomechanical testing and histology

The biomechanical tests were conducted with a rheometer (MCR302e; Anton Paar, Graz, Austria) in combination with RheoCompass software (Anton Paar). To ensure comparability across other tissues previously tested, the methodology followed the process defined in our previous manuscripts [1, 2]. The tissues were loaded into the rheometer under an axial preload of 0.1 N to ensure adhesion of the contact surfaces and then given 100 s for residual stress relief. To reduce slippage, the contact surface of the measuring tool and the base plate were covered with waterproof sandpaper (Type P120 according to the Federation of European Producers of Abrasives; Fig. 1D). A peak angular shear strain of 3% at 3 Hz, with a continuous 0.1 N compression force, was applied for a total of 50 cycles. The rheometer was calibrated using phosphate-buffered saline (PBS), and all tests were conducted in PBS at 20 °C.

Immediately following the biomechanical tests, all samples were placed in 4% paraformaldehyde for full fixation and further processed according to a standardized histology protocol. One H&E-stained slide was prepared in the circular plane of the punched tissue cylinder and jointly analyzed by two investigators (J.Z. and B.O.) under a light microscope by consensus (Olympus BX51, Olympus Corporation, Tokyo, Japan). For the histological analyses, only the case number was known to the investigators to avoid biases, such as those that could arise from knowing the PMI when screening the tissues for signs of autolysis. The following features were assessed: (I) The pathological state of the tissue, classified as healthy (defined as the absence of pathological signs), low-grade fatty (adipocyte content of the assessed slide < 50% of the entire section), high-grade fatty (adipocyte content of the assessed slide ≥ 50% of the entire section), or cirrhotic; (II) The presence of blood congestion (yes or no); (III) The presence of autolysis (yes or no). Representative images were digitized using the Aperio CS2 scanner and the Aperio ImageScope software (both Leica Biosystems, Nussloch, Germany).

### Data analysis 

The storage modulus (G’), loss modulus (G’’), and complex shear modulus (G*) were determined. The data were entered into Microsoft Excel Version 16.74 (Microsoft Corporation, Redmond, USA). Statistical analyses and data visualization were performed using GraphPad Prism version 9 (GraphPad Software, La Jolla, USA). The Kolmogorov-Smirnov test was used to assess the Gaussian distribution of the data. Depending on the Gaussian distribution, either ordinary one-way ANOVA tests, including Tukey’s multiple comparisons, or Kruskal-Wallis tests, including Dunn’s multiple comparisons, were applied when at least three groups were present. For cases with only two groups, parametric t-tests and nonparametric Mann-Whitney U-tests were applied for parametric and nonparametric data, respectively. Accordingly, Pearson’s or Spearman’s correlation coefficients were reported following two-tailed analyses for parametric and nonparametric correlations. P-values ≤ 0.05 were considered statistically significant.

Only samples taken from adult cadavers (minimum age at death: 18 years) were included in the statistical analyses to avoid data bias due to developmental inhomogeneity of the overall sample. Therefore, the data from the four pediatric samples included in this study are only reported descriptively. The following statistical analyses were performed: Firstly, the mechanically tested samples were microscopically evaluated and the rheological properties compared per pathological grade. This included a comparison of the liver weight, age at death, sex and PMI among the four different subgroups. Secondly, the dependencies of the rheological properties of healthy and combined fatty samples on liver weight, age at death, sex and PMI as well as the histologically diagnosed signs of autolysis and venous congestion were investigated.

## Results

### Comparisons of healthy, fatty and cirrhotic liver samples

**Fig. 2:**
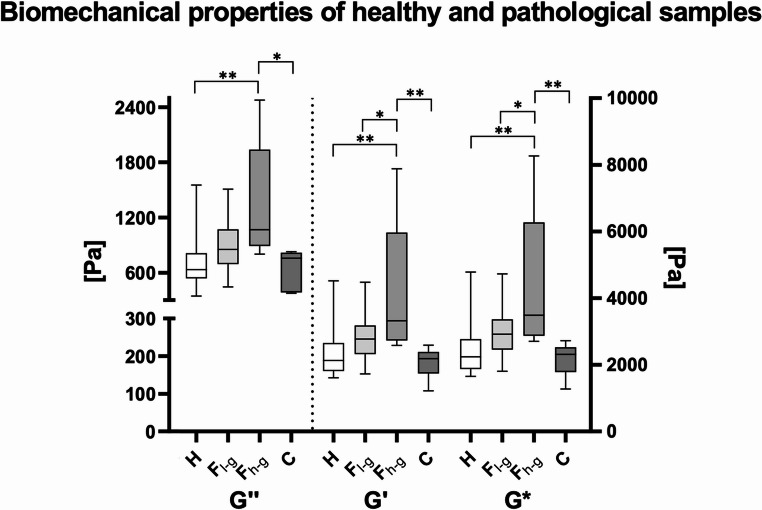
The rheological properties of healthy (H), low-grade fatty (F_l−g_), high-grade fatty (F_h−g_) and cirrhotic samples (C) are depicted. The outlines of the boxes indicate the 25% and 75% percentile, the solid black horizontal line the median. Whiskers indicate the minimum and maximum. The dotted line separates left and right y-axis. G’, storage modulus; G’’, loss modulus; G*, complex shear modulus; *, p-value ≤ 0.05; **, p-value ≤ 0.01

**Fig. 3:**
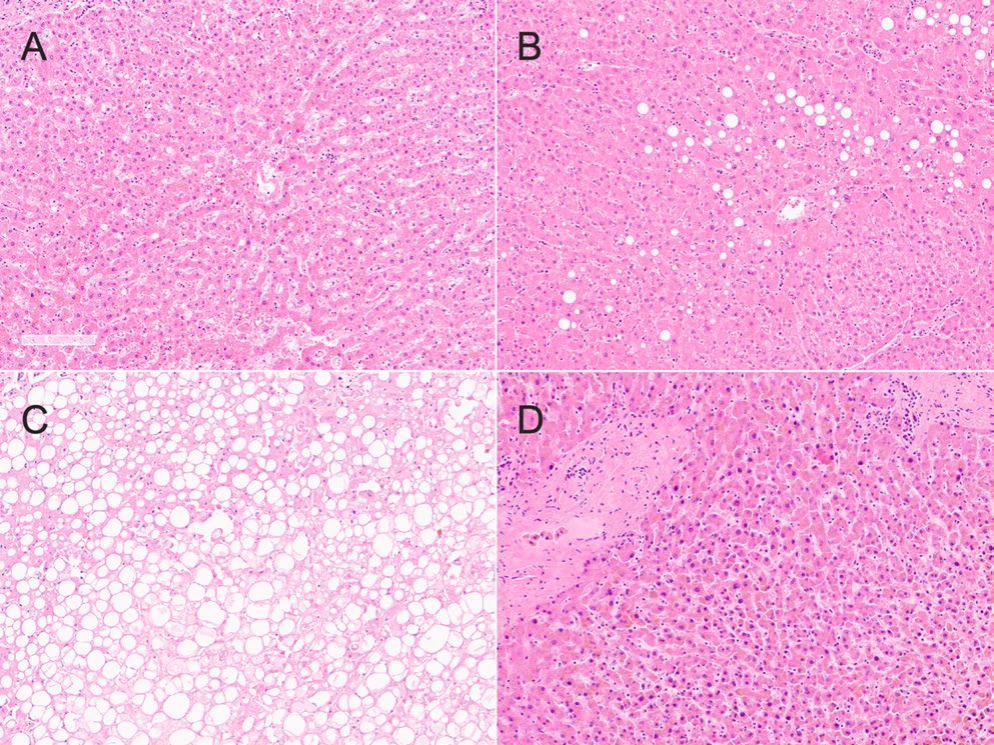
Microscopic images of the tested samples are shown. **(A)** Healthy sample (*n* = 27, including three pediatric cases). **(B)** Low-grade fatty sample (*n* = 14, including one pediatric case). **(C)** High-grade fatty sample (*n* = 6). **(D)** Cirrhotic sample (*n* = 7). All images depicted at 8x magnification. Scale bar: 150 micrometers

High-grade fatty liver samples showed a significantly higher G’ compared to all other subgroups (ANOVA; F(3, 46) = 7.72; healthy samples: *p* < 0.01; low-grade fatty samples: *p* = 0.02; cirrhotic samples: *p* < 0.01). The G’’ of high-grade fatty liver samples was significantly higher compared to both healthy and cirrhotic samples (Kruskal-Wallis; H(3) = 14.07; healthy samples: *p* < 0.01; cirrhotic samples: *p* = 0.04), but not compared to low-grade fatty samples. High-grade fatty liver samples showed a significantly higher G* compared to all other subgroups (ANOVA; F(3, 46) = 7.76; healthy samples: *p* < 0.01; low-grade fatty samples: *p* = 0.02; cirrhotic samples: *p* < 0.01). A graphical depiction of the measured rheological properties of the four subgroups is given in Fig. 2. Microscopic images of exemplary samples of the four subgroups are shown in Fig. 3. Based on the results of the group comparisons, Receiver Operator Characteristic (ROC) curves with a 95% confidence interval were plotted between the fatty (combined low- and high- grade) and the remaining samples (healthy and cirrhotic) to determine the diagnostic ability of the biomechanical test to detect fatty degeneration of the sample. According to ROC curve analyses, fatty samples could be discriminated from healthy and cirrhotic samples with good diagnostic ability based on the here analysed rheological properties **(**Table 1**)**. The data of the four pediatric cadavers is shown in Table 2. All of the rheological properties of the pediatric samples were within the range of the adult samples.

**Table 1:** The cut-off values of the investigated rheological properties with the best positive likelihood ratios of the ROC curve analyses are depicted. AUC, area under the curve; CI, confidence interval; G’, storage modulus; G’’, loss modulus; G*, complex shear modulus

Rheological property	AUC	Cut-off value [Pa]	Sensitivity [%]	95% CI [%]	Specificity [%]	95% CI [%]	Likelihood ratio
G*	0.786	> 3510	26	11.81–48.79	97	83.81–99.83	8.16
**G’**	0.784	> 3355	26	11.81–48.79	97	83.81–99.83	8.16
**G’’**	0.795	> 1041	37	19.15–58.96	97	83.81–99.83	11.42

**Table 2:** The data of the four pediatric cases is shown. F_l−g_, low grade-fatty sample; G’, storage modulus; G’’, loss modulus; G*, complex shear modulus; PMI, post-mortem interval

Age	Sex	Pathology	Liver weight [g]	Autolysis	Blood congestion	PMI [h]	G’ [Pa]	G’’ [Pa]	G* [Pa]
2 month	f	N	178	No	Yes	63	1728	410	1776
2 yrs.	m	F_l−g_	405	No	No	64	2007	461	2059
5 yrs.	m	N	586	No	Yes	112	2541	727	2643
6 yrs.	m	N	696	No	No	108	1648	408	1698

**Fig. 4:**
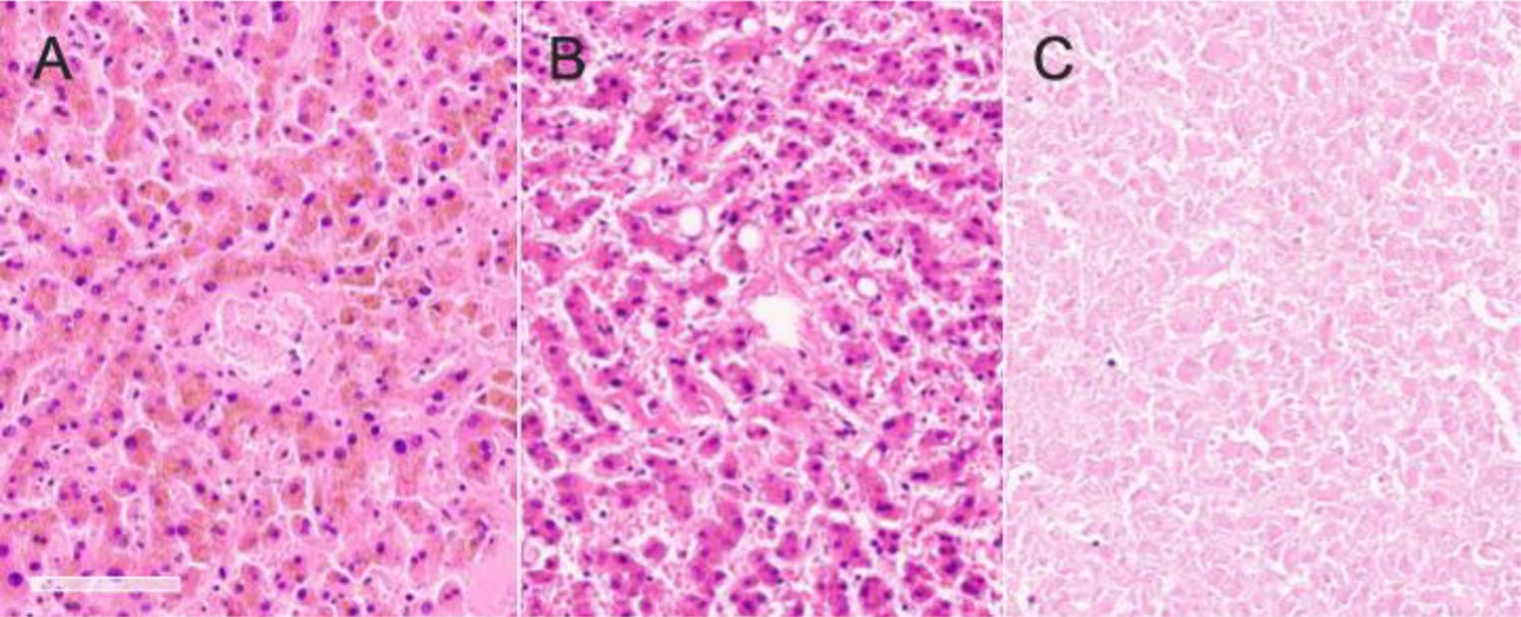
Representative images of microscopically-based assessments of autolysis and blood congestion are depicted. **(A)** Sample with blood congestion. **(B)** Sample without signs of blood congestion. **(C)** Autolytic sample. All images depicted at 20x magnification. Scale bar: 100 micrometers

The liver weight of high-grade fatty samples was significantly higher compared to healthy liver samples (ANOVA; F(3, 46) = 5.41; *p* < 0.01). Healthy, fatty and cirrhotic samples were statistically indistinguishable in regard to both age at death (ANOVA; F(3, 46) = 0.04; *p* ≥ 0.98) and PMI (ANOVA; F(2, 39) = 2.35; *p* ≥ 0.09; high-grade fatty liver samples not included due to low number of comparisons with *n* = 4). The portion of (at least partly) autolytic samples of healthy, low-grade fatty, high-grade fatty and cirrhotic samples were 46%, 38%, 53% and 57%, respectively. The portion of samples with blood congestion of healthy, low-grade fatty, high-grade fatty and cirrhotic samples were 58%, 69%, 63% and 86%. Representative images of the microscopic assessments of autolysis and blood congestion are shown in Fig. 4.

### Rheological properties of healthy and fatty samples

The G’’ of autolytic healthy liver samples of 828 ± 284 Pa (*n* = 11) was significantly higher compared to non-autolytic healthy liver samples with 572 ± 178 Pa (*n* = 13) **(**Table 3**)**. In fatty liver samples the G’’ of autolytic (*n* = 6) and non-autolytic (*n* = 13) samples was statistically similar with 979 ± 313 Pa and 1054 ± 535 Pa, respectively. The G’’ of congested healthy liver samples of 619 ± 205 Pa (*n* = 14) was significantly lower compared to samples free of blood congestion with 788 ± 310 Pa (*n* = 10) **(**Table 3**)**. The same was true for fatty samples, for which the G’’ of congested samples was 800 ± 187 Pa (*n* = 12) and for samples free of blood congestion 1425 ± 552 Pa (*n* = 7) **(**Table 3**)**. Concomitantly, the G’ and G* of congested fatty liver samples of 2623 ± 542 Pa and 2744 ± 563 Pa was significantly lower when compared to uncongested fatty liver samples with 4294 ± 1852 Pa and 4525 ± 1931 Pa, respectively **(**Table 3**)**.

**Table 3:** The results of the statistical analyses are shown for the dependencies of the rheological properties on several parameters for healthy (H) and fatty (F, including both high- and low-grade fatty) samples. Cirrhotic samples were excluded due to low number of pairs. G’, storage modulus; G’’, loss modulus; G*, complex shear modulus; p, p-value (*, significant); P(r); pearson’s correlation coefficient; U, Mann-Whitney-U (Mann-Whitney U test); S(r), spearman’s correlation coefficient; t, t-value (T-test)

		G’		G’’		G*	
**Liver weight**	H	P(r)=−0.01	*p* = 0.96	S(r) < 0.01	*p* = 0.99	P(r)=−0.02	*p* = 0.93
	F	S(r) = 0.36	*p* = 0.13	S(r) = 0.22	*p* = 0.36	S(r) = 0.36	*p* = 0.13
**Age at death**	H	P(r)=−0.24	*p* = 0.27	S(r)=−0.12	*p* = 0.59	P(r)=−0.24	*p* = 0.26
	F	S(r)=−0.01	*p* = 0.97	S(r) = 0.03	*p* = 0.91	S(r)=−0.03	*p* = 0.89
**Sex**	H	U = 50	*p* = 0.82	U = 43	*p* = 0.49	U = 50	*p* = 0.82
	F	U = 32	*p* = 0.58	U = 27	*p* = 0.32	U = 31	*p* = 0.52
**Autolysis**	H	t = 1.07	*p* = 0.30	t = 2.69	*p* = 0.01*	t = 1.22	*p* = 0.23
	F	U = 32	*p* = 0.58	t = 0.31	*p* = 0.76	U = 33	*p* = 0.64
**Blood congestion**	H	t = 0.99	*p* = 0.33	U = 33	*p* = 0.03*	t = 1.06	*p* = 0.30
	F	t = 2.97	*p* = 0.01*	t = 3.63	*p* < 0.01*	t = 3.04	*p* = 0.01*

### Post-mortem interval

The rheological properties of healthy (n = 23), fatty (n = 16) and cirrhotic (n = 7) liver samples showed no significant correlation with the PMI (healthy: G’: P(r)=−0.06, p = 0.80; G’’: P(r) = 0.33, p = 0.13; G*: P(r)=−0.02, p = 0.92; fatty: G’: S(r)=−0.07, p = 0.81; G’’: S(r) = 0.14, p = 0.59; G*: S(r)=−0.05, p = 0.86; cirrhotic: G’: P(r) = 0.06, p = 0.90; G’’: P(r) = 0.07, p = 0.89; G*: P(r) = 0.06, p = 0.90). A significant positive correlation between the PMI and G’’ was shown for healthy samples, if only samples without signs of blood congestion were included (P(r) = 0.76; p = 0.02; n = 9). A correlation between G’’ and PMI depending on the status of blood congestion could not be checked for fatty and cirrhotic samples due to the low number of pairs. Healthy autolytic samples (*n* = 10) revealed a significantly higher PMI (291 ± 44 h) compared to samples without histological signs of autolysis (171 ± 81 h; t U = 19; *p* < 0.01, *n* = 13).

## Discussion

This study aimed to objectively investigate the factors that impact the biomechanical assessment of the liver at autopsy, including factors known from forensic files, diagnosed during the autopsy, and identified through histology after the autopsy. Moreover, it intended to investigate the use of human liver samples taken during routine forensic autopsies for biomechanical time since death estimation.

### Fatty infiltration, autolysis and blood congestion influence liver biomechanics – liver weight, age at death and sex showed no significant trends

The results revealed that fatty infiltration profoundly influences the rheological properties of liver tissue, increasing G’, G’’, and G* (Table 1). This suggests that biomechanical time since death estimations using liver tissue must be accompanied by histological analysis to accurately differentiate between fatty and non-fatty samples, thereby avoiding biases based on the pathological state of the tissue. The results of the ROC curve analyses indicate that rheometry can effectively discriminate between fatty and non-fatty liver samples with good diagnostic ability, underscoring the substantial biomechanical differences between them (Fig. 2). Theoretically, rheometry tests could serve as a diagnostic tool to detect fatty liver samples. The main advantage over histological analysis is the time efficiency of the method: rheological testing takes about two to three minutes in total and could complement the macroscopically diagnosed fatty liver, for example, in autopsies performed immediately in homicide cases. In theory, the handling and testing time required for rheological assessment increases the PMI, potentially affecting the tissue’s rheological properties. In contrast, immediate immersion of the sample in formaldehyde effectively “freezes” the organ’s structure. However, given that the handling and testing duration for the rheological method described here is only a few minutes, its impact on the measured properties is considered negligible. However, histological analysis remains the gold standard for detecting fatty degeneration of liver tissue, as it provides more comprehensive information beyond the binary classification of fatty versus non-fatty samples.

Both histological signs of autolysis and blood congestion significantly altered the G’’ compared to non-autolytic and uncongested samples, respectively. The G’’ refers to the energy lost under deformation, which describes the viscous behavior of the tissue [3]. Autolysis, the enzymatic breakdown of cellular material, leads to the disintegration of originally well-structured cellular arrangements into an unstructured mush-like paste, thereby increasing the viscous component of the overall sample. However, in this study, the change induced by autolysis was observed only in healthy samples and was absent in fatty samples. One possible explanation is that the increased G’’ due to fatty infiltration masks the subtle autolytic changes observed in healthy samples. Additionally, the low sample number of *n* = 6 for autolytic fatty samples should be noted.

Blood congestion resulted in a significant decrease in the G’’ in both healthy and fatty samples, which might seem counterintuitive since one would expect an increase in the viscous portion due to the increased blood volume. However, the blood is primarily contained within the blood vessels, thereby enhancing the stability of the vascular network within the overall sample and reducing the portion of energy dissipated as heat. While blood congestion affected only the G’’ in healthy samples, it significantly altered the G’ and G* in fatty liver samples as well. This indicates that blood congestion appears to offset the biomechanical changes induced by fatty infiltration.

The rheological properties of healthy and combined fatty liver samples did not correlate with liver weight at autopsy, age at death, or sex of the deceased. Therefore, none of these factors can serve as indicators for a particular biomechanical state of the tissue. For example, heavy livers, which are often associated with fatty infiltration or blood congestion, are not necessarily related to higher G’ or G’’. Surprisingly, the here measured rheological properties of cirrhotic samples were statistically similar to both healthy and low-grade fatty samples. From autopsy experience, cirrhotic samples seem to be stiffer than healthy and fatty samples.

### Time since death estimation

This study explored the use of tissue mechanics for forensic time since death estimation using human liver samples retrieved during routine forensic autopsies. The cadavers were transferred to a cooling unit with an ambient temperature of 4 °C within a few hours after death and were stored for an average of eight days. Among the rheological properties investigated, the G’’ of healthy liver samples without histological signs of blood congestion significantly increased with increasing PMIs. However, this was only among 6 subjects, and thus prospective studies should confirm this finding. Nonetheless, the finding aligns with the previously discussed explanation for autolytic changes. The positive correlation between the G’’ and PMI was absent in healthy liver samples with histological signs of blood congestion and in fatty liver samples. This indicates that both blood congestion and pathological changes may mask the PMI-related variations in the G’’ within the tested PMI interval, as described earlier.

From a practical perspective, a concomitant histological investigation of liver samples is essential for accurate biomechanical time since death estimations in each case. The significant change in the loss modulus (G’’) of healthy liver samples without histological signs of blood congestion demonstrates that, under routine storage conditions up until autopsy, the biomechanical state of the human liver changes significantly. Therefore, from a biomechanical perspective, storage times should be minimized. However, it is unclear if the subtle changes detected in this study would be relevant when considering the routine manual subjective assessment during autopsy. The results of this study are valid for tissues that are cooled at 4 °C between death and the biomechanical test. Temperature is a significant confounder when assessing the rheological properties of biological tissues post-mortem [2]. Therefore, this study should be repeated using different storage temperatures within a range relevant to forensic practice, such as from freezing point to body temperature. This would allow for interpolation of most rheological properties across various storage temperatures, without the need to test every possible temperature in between.

From a legal perspective, performing an autopsy requires authorization, which in Germany is granted by the public prosecutor’s office or confirmed by relatives. This decision typically takes approximately two days in standard cases. Consequently, the availability of samples with a PMI of less than two days is limited. Moreover, the need to cool the cadaver between admission and autopsy to prevent degradation makes it challenging to explore other storage temperatures for this method in standard cases. Irrespective of legal concerns, immediately performed autopsies of uncooled cadavers - such as in suspected or confirmed homicide cases - could be a valuable resource to establish the method, provided that the time of death and ambient temperature are known.

### Value beyond forensic science

In order to be able to check the quality of computational models, experimental data on the rheological properties of human liver are needed beyond forensic practice for various applications, including medically related finite element method (FEA) analyses, virtual reality simulators, and injury biomechanics in other fields [4–8]. However, there are only a few ex-vivo biomechanical investigations of human liver tissue to date [4, 9–14]. The scarcity of data is primarily due to the limited availability of human liver tissues.

One study included samples from 20 different cadavers but only performed histology to assess the main fiber orientation rather than the pathological state of the organs [4]. Another study investigated samples from 19 different individuals, all of whom had liver diseases, predominantly carcinomas, thus the study did not represent the healthy state [15]. Similarly, another study included only four healthy cadavers out of a total cohort of 14 cadavers [10]. In the remaining studies, samples were taken from only one to seven different cadavers [9, 11, 13, 14] and in one study the number of samples was not stated [12]. One ex-vivo study demonstrated that liver fibrosis was associated with an increase in the elastic modulus of the tissue [15]. In contrast, this study showed that the G’ of cirrhotic samples was statistically similar to that of healthy samples, with increases primarily related to fatty infiltration of the tissue. Furthermore, the G’ and G’’ of healthy samples measured in this study are within the order of magnitude reported for healthy samples in an in-vivo investigation using multifrequency magnetic resonance elastography on 17 volunteers [16]. Moreover, the presented pediatric rheological properties in this paper were within the order of magnitude of the adult ones, however, only a small number of samples was available.

## Conclusions

Fatty degeneration of the liver significantly increases the storage, loss, and complex shear moduli compared to healthy and cirrhotic livers. Additionally, autolysis and blood congestion affect the rheological properties of liver tissue, whereas liver weight, age at death, and sex had no discernible impact. The minor changes in rheological properties observed within the average tested PMI of eight days and at 4 °C storage suggest that liver tissue holds promise for biomechanical time since death estimations.

### Limitations

Firstly, this study was limited by its sample size although it comprised the highest number of biomechanically analysed human liver samples to date. Secondly, potential biomechanical changes occurring between death and the minimum PMI of 42 h might have been missed due to the legal time required to authorize and schedule the autopsy. Thirdly, the temperatures during the uncooled period before refrigerated storage in the morgue could not be controlled and likely varied, which may have influenced the results. However, a rectal temperature of at least 30 °C upon arrival at the morgue was used as an inclusion criterion to minimize the confounding effects of delayed cooling on the tissue biomechanics. Fourthly, in three cases where the exact time of death was unknown, the time the body was found was used as a proxy for the time of death. In these cases, the rectal temperature upon arrival at the morgue was above 36 °C, suggesting a rather short time interval between the actual time of death and the time the body was found, especially, as all three cases died from non-infectious diseases. Lastly, comparisons of the here obtained rheological properties to macroscopic features of the liver were omitted as histology was defined as the gold standard for pathological diagnoses.

## Data Availability

The data is available on request.
